# Health Implications of Drinking Water Salinity in Coastal Areas of Bangladesh

**DOI:** 10.3390/ijerph16193746

**Published:** 2019-10-04

**Authors:** Rishika Chakraborty, Khalid M. Khan, Daniel T. Dibaba, Md Alfazal Khan, Ali Ahmed, Mohammad Zahirul Islam

**Affiliations:** 1 Department of Environmental and Occupational Health, School of Public Health, Indiana University Bloomington, IN 47405, USA; rchakra@iu.edu; 2 Tennessee Clinical and Translational Science Institute, University of Tennessee Health Science Center, Memphis, TN, 38105, USA; ddibaba@uthsc.edu; 3 Matlab Health Research Centre, International Centre for Diarrhoeal Disease Research, Bangladesh (ICDDR,B), Dhaka 1212, Bangladesh; fazal@icddrb.org; 4 International Centre for Diarrhoeal Disease Research, Dhaka 1212, Bangladesh; mzislam@icddrb.org (A.A.); aliahmed2007@gmail.com (M.Z.I.)

**Keywords:** water salinity, coastal Bangladesh, health effects, hospitalization

## Abstract

Coastal areas in South Asian countries are particularly vulnerable to elevated water salinity. Drinking water salinity has been found to be associated with cardiovascular diseases (CVD), diarrhea, and abdominal pain. Our study aimed to find if excess drinking water salinity was associated with increased hospital visits with an array of health effects in coastal sub-districts of Bangladesh. A cross-sectional study was conducted with 157 participants from three coastal sub-districts. A face-to-face interview was conducted to collect salinity exposure and hospital visit data. Water samples were collected from both drinking and other household water sources for the measurement of salinity and total dissolved solids (TDS). CVD, diarrhea, and abdominal pain related hospital visits were found to be significantly associated with high water salinity and TDS. Households exposed to high salinity demonstrated a higher frequency of hospital visits than the low salinity-exposed households. People exposed to high salinity seemed to lack awareness regarding salinity-inducing health effects. Water salinity is a public health concern that will continue to rise due to climate change. Therefore, raising awareness about the health risks of water salinity is essential for the government to frame policies and mitigation strategies to control this emerging threat.

## 1. Introduction

Water salinity has been identified as an increasing public health concern affecting thousands of households every year. Salinity intrusion occurs significantly in coastal areas of Asian countries, such as Bangladesh, China, and Vietnam, but has also been reported in California, Brazil, and the Netherlands [1]. Exposure to a high level of saline via drinking water in coastal populations has led to increased cardiovascular and other diseases. Climate change, which leads to sea level rise and exacerbates cyclones and storm surges, is one of the primary drivers of water salinity [2]. Over extraction of ground water and construction of canals and dams are some human activities that aggravate the situation [3,4,5,6,7]. 

The geomorphology of Bangladesh has always made the country vulnerable to natural hazards such as storm surges, cyclones, inundation, and seawater intrusion. The southern coastal regions of Bangladesh are only 1m to 3m above mean sea level [3], which has resulted in seawater contamination of its drinking water [7]. The wide river mouths make drinking water prone to excessive salinity, as they allow the seawater to flow with great rapidity further inland. Drinking water salinity is expected to further increase due to climate change and the consequent rise in sea level [2]. In addition to natural causes for increased salinity in drinking water in Bangladesh, there are several anthropogenic reasons at play. One such is the construction of Farakka Dam in 1975 on the Ganges River. The freshwater diversion upstream caused a change in the natural water flow in the downstream. The dry months from December to May saw a significant reduction in flow, which resulted in increased river salinity [8]. Other significant contributing factors are shrimp farming, construction of coastal polders, and over- extraction of groundwater [3,4,5,6,9].

Consumption of excess saline in drinking water has been linked to a variety of health effects. Salt in drinking water is generally found in low levels (20 mg/L) and is considered a negligible contributor to daily salt intake. WHO, therefore, does not have a health-based standard, but an aesthetic guideline value of 200mg/L [10]. However, several studies identified the association between excessive salinity in drinking water with increased risk of hypertension [11,12]. Drinking water salinity has also been linked to risk of preeclampsia and gestational hypertension [13]. In addition, there are reports on association with infant mortality, cholera outbreaks, and skin and diarrheal diseases [14,15]. Proxies of water salinity are total dissolved solids (TDS) and electrical conductivity. While US Environmental Protection Agency (EPA) has set a secondary standard (non-mandatory guideline) value of TDS at 500 mg/L, risk of heart diseases have been associated with increased TDS [16,17]. Thus, increased water salinity may lead to several adverse health effects. This may not only increase the hospital visit rates of the affected population, but may also drive up the healthcare costs.

The primary aim of this study was to investigate the association between salinity exposures with hospital visits for a variety of health effects in Bangladesh. Both salt content and TDS were measured as exposure variables in this study. The secondary aim of the study was to assess the level of awareness regarding salinity exposure in a sample of exposed households. Populations in coastal Bangladesh are exposed to such extremes of salt via drinking water that it overshadows their dietary salt consumption. Bangladesh has a standard value of drinking water salinity of 600 mg/L [18,19]. The high salinity naturally present in the coastal waters makes the inhabitants vulnerable to a host of health effects. Despite this increasing public health issue, the government has largely neglected its consequences. Data generated by our study may help high water salinity-affected countries to take preventive measures and develop appropriate policies and community action plans to mitigate this crisis. 

## 2. Materials and Methods 

### 2.1. Study Design and Participants

We conducted a population-based cross-sectional study between September 2016 and January 2017 in three rural coastal sub-districts located in south and southwest regions of Bangladesh. Based on published data and unpublished water salinity data generated by our local research collaborators from the International Center for Diarrheal Disease Research Bangladesh (ICDDR,B) these three sub-districts Mathbaria (population 264 thousands) and Zianagar (population 72 thousands) under Perojpur district (average electrical conductivity 5380.0 µS/cm) and Mongla (149 thousands) under Bagerhat district (average electrical conductivity 11,370.4 µS/cm) were selected as study areas since they were very well-known for high drinking water salinity [20,21]. Interviewers were field research assistants working at ICDDR,B who had prior knowledge about the study areas. Our collaborators from ICDDR,B had a list of households who previously participated in other ICDDR,B public health projects. From the list of around 700 households, we randomly selected 60 households each from the 3 selected study areas, that met the eligibility criteria such as a physically active and married head of the household aged 19–60 years, residing in those specific rural communities for more than 3 years, and having at least one physically active child in the family with ≥10 years of age. We subsequently invited the head of the households to participate in a questionnaire survey and provide a water sample from the source of drinking water and a sample of water used for other purposes at the household such as bathing and cooking. Out of 180 invited participants, 157 finally agreed to participate in the study. Data entry was done by a trained data analyst working in ICDDR,B and the quality of data was reviewed by a senior biostatistician from the same organisation. The study protocol was approved by the institutional review board (IRB) of Indiana University Bloomington (IRB protocol number: 1511886945) on 4 January 2016 and the ethics committee of the ICDDR,B. All participants gave written informed consent before participating in the study activities. 

### 2.2. Questionnaire Survey 

We conducted a face-to-face interview with a structured questionnaire to collect data on our primary outcome, which is the frequency of hospital visits by the participant or any family member. We also collected data on sociodemographic characteristics, water use, and perception of health effects of water salinity. Height and weight of the participants were also recorded by our study interviewer to compute body mass index (BMI) using a measuring tape (cm) and a weighing scale (kg), respectively. The health outcomes we measured were CVDs, diarrhea, abdominal pain, gastric ulcer, dysentery, skin diseases, and typhoid. CVDs were defined as heart attacks, stroke, heart failure, arrhythmia, and heart disease, as well as its associated risk factor, hypertension (defined as blood pressure > 140/90 mmHg) since it is highly prevalent in Bangladesh [22]. Diarrhea was defined as the passage of 3 or more loose or liquid stools per day, or more frequently than is normal for the individual. Abdominal pain was considered as chronic pain in the abdomen, gastric ulcer was defined as a burning sensation in the upper abdomen, and dysentery was defined as diarrhea with visible red blood, while skin diseases included discoloration, rashes, allergy, and infection. 

### 2.3. Water Sample Collection and Analysis

Two water samples for primary (i.e. source of drinking water) and secondary (i.e. source of water for cooking and bathing) sources for household use of water were collected in a 250 ml plastic bottle by the study interviewer right after the end of the interview. Tubewell water was mainly used for drinking purposes, while pond water was used for other (cooking and bathing) purposes. A water sample collection guideline was prepared based on standard field procedures and the staff was trained accordingly [23,24]. After the water samples were collected, they were preserved in the field. A temporary lab facility was established in ICDDR,B for the water sample testing. The samples were subsequently sent to ICDDR,B for measuring water salinity using a HACH sensION5 Electric Conductivity meter and Platinum conductivity cell (model 5060, HACH, Loveland). Salinity measure was reported in terms of Electrical Conductivity (EC) in µS/cm, Salinity (SAL), and TDS in milligrams per liter (mg/l) at 25°C. Although samples were not at the reference temperature during collection and measurement, the meter had the ability to automatically calculate conductivity adjusted for 25 °C [20]. 

For analysis, tubewells were delineated between shallow and deep tubewell by a cutoff point of 150 meters (~500 feet) [25]. In the set-up lab facility, the water samples were tested following the standard procedure recommended in the HACH sensION5 Electric Conductivity meter guideline. Each morning, a 3-point calibration was done with the 147 µS/cm, 1413 µS/cm, and 12.88 mS/cm calibration liquids. After each 5 testing, the probes were checked randomly to see their calibration consistency. Each sample was tested 3 times and then the average value recorded. 

### 2.4. Statistical Analyses

All participants were categorized into two groups (high vs low salinity exposure) using the median salinity level (172 mg/L) of the primary source of water as the cut off value. We used the median drinking water salinity level as cut off since it is one of the standard statistical approach to categorize salinity exposure and it is also closer to the WHO guideline value of 200mg/L, and therefore has a wider generalizability. We compared demographic characteristics between the high and low salinity exposure groups using descriptive data presented as frequency (%) or means ± standard deviations. Initial bivariate analyses were conducted to compare differences in proportions using Chi-square test and mean differences using independent t-tests. Statistical significance was defined as p < 0.05 and all hypothesis tests were 2-sided. Chi–square test or Fisher’s exact test was performed to assess if the two water salinity exposure groups differed in the frequency of hospital visits by all the family members of a household in the past twelve months. Furthermore, participants were categorized into high and low TDS exposure using the average TDS value (primary source of drinking water only) and then the two TDS exposure groups were compared for past twelve months of hospital visits, an indicator of health effects of poor drinking water quality. We constructed multivariate logistic regression models to examine the associations between quartiles of water salinity and hospital visits with various common health effects reported among rural residents using the lowest quartile as the reference category. Potential confounding variables selected for initial inclusion in the logistic statistical models were those documented as socio–economic risk factors of various common water-associated diseases. We examined whether these covariates changed the estimated associations between exposure and effects; variables were retained in the model if there was any substantial change (i.e. > 10%) in the association of interest. Community perceptions on water salinity were computed in the form of frequency and compared by Chi-square tests between high and low salinity affected participants using the median salinity level for the primary source of drinking water. All statistical analyses were performed using SAS 9.4 (SAS Institute, Cary, NC, USA).

## 3. Results

### 3.1. Socio–Demographic Characteristics

The socio–demographic characteristics of all participants are shown in Table 1. Drinking water salinity was categorized based on the median (172 mg/L) in the data. A total of 157 participants were recruited in the study, 79 from high (≥ 172 mg/L) and 78 from low (<172 mg/L) salinity areas. Only the head of the household for each household was interviewed, who were predominantly male. A higher percentage of the population in the low salinity area had attained an educational degree beyond the elementary level, however, this difference was not significant. No significant differences were found in the BMI, annual income, and smoking behavior between the high salinity exposed and low salinity exposed groups. A significantly higher amount of salinity and TDS was found in the drinking water in high salinity areas. However, the salinity and TDS concentrations for other use (pond water) were lower in the high salinity areas. This may be explained by the fact that pond water generally has lower salinity levels than tubewell water throughout the year [26].

### 3.2. Hospital Visit Frequency

Hospital visits due to different health effects caused by high salinity (≥172 mg/L) and associated TDS in drinking water, stratified by high and low salinity areas have been outlined in Table 2. Only cases that reported more than three hospital visits in a year due to the same health effect was considered in the table. A significant association was found between hospital visits due to high salinity and TDS in drinking water with CVD, diarrhea, and abdominal pain. The general trend showed population from high salinity areas had a higher frequency of hospital visits than people from low salinity areas. 

The odds ratio (OR) for hospital visits for CVD, diarrhea, and abdominal pain also demonstrated consistently higher risk for the fourth quartile of drinking water salinity when compared with the lowest quartile after accounting for the potential covariates’ education and annual household income. The third quartile readings for CVD and diarrhea are inconsistent with the general trend. However, these associations were not statistically significant (Table 3).

### 3.3. Perceptions on Water Salinity

**Figure 1:**
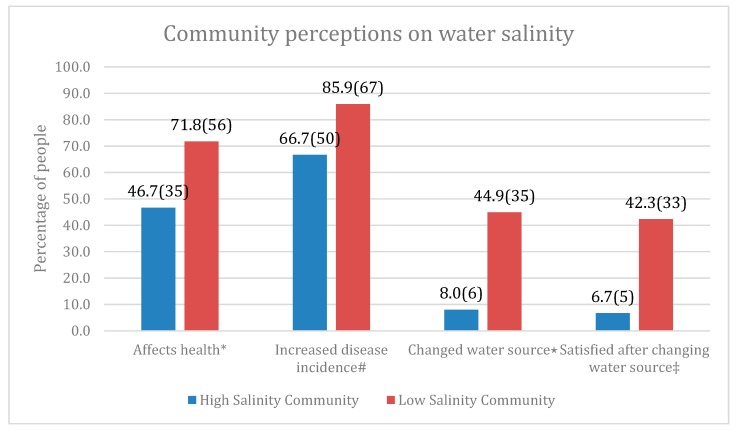
Perceptions on water salinity from high and low salinity communities. Note: Bars shows %(*n*) values. * *p* value = 0.002; # *p* value < 0.0001; ⋆ *p* value = 0.003; ‡ *p* value = 0.005.

Based on the median data, participants from low water salinity area were found to have a significantly higher awareness about the health effects of drinking excess saline in water than participants from high salinity area. 71.8% of the participants from low salinity area believed that drinking water salinity has a negative effect on health, while more than half the population from high salinity area did not think salinity posed a health threat. 85.9% of the participants from low salinity area thought there was an increase in disease incidence due to increased salinity over the last 15 years, as opposed to only 66.7% from the high salinity area. It was seen that 76% of the participants from high salinity area did not change their water source even after disease incidence. A substantial number of people (42.3%) from the low salinity area were found to be satisfied after changing their water source. These differences were statistically significant at *p* < 0.01 (Figure 1). Data from 75 people were obtained from the high salinity area and 78 people from low salinity area. 

## 4. Discussion

In our study, we reported an increased frequency of hospital visits for several health effects in high salinity areas of coastal Bangladesh. We found significant differences in hospital visit rates between high and low salinity exposed groups for CVD, diarrhea, and abdominal pain. With increasing quartiles of water salinity, the hospital visit rates for CVD, diarrhea, and abdominal pain were found to increase. Our study also found a lack of awareness among people from high salinity areas regarding drinking water salinity. The WHO based aesthetic guideline value for salt in drinking water does not take into account the extreme levels of salt naturally occurring in coastal Bangladesh, which has detrimental health effects. 

Our study reported increased hospital visits for CVD in people from high salinity areas, which is consistent with several previous studies that reported a positive association between drinking water salinity and hypertension, which is one of the strongest risk factors for CVD. A cohort study in Bangladesh reported a direct relationship between drinking water salinity and blood pressure, which also found that lowering sodium intake in drinking water lowered both systolic and diastolic blood pressure [7,27,28]. A study in healthy pregnant women also found that those who drank water from tube wells or pond water, which has higher sodium content, had significantly higher blood pressure (both systolic and diastolic) than those who drank rainwater [28]. In another recent study, an increased risk of hospital visits due to hypertension in high salinity exposed areas was observed [29]. A meta–analysis also suggested an association between risk of hypertension and water salinity [11]. However, a small number of studies reported otherwise. A study in Chicago found that while the diastolic blood pressures of males and females were higher in the participants exposed to high salinity, the systolic blood pressures were not significantly different between the high and low sodium communities [30]. Another study in Arizona, USA found no association between saline levels in water and systolic or diastolic blood pressure [31]. Therefore, while some studies did not report association between drinking water salinity and hypertension, studies that are more recent have demonstrated overwhelming evidence of this relationship. Increase in blood pressure can lead to a myriad of heart diseases such as angina, heart failure, and heart attack, and can also cause stroke. Considering the relatively younger age of the household heads in our study, from our results we may assume that there was a higher risk of early CVD onset in the people from high salinity areas than the low salinity areas. However, this has not been addressed in our study and further research in this area is vital. Since rising water salinity levels directly affect hypertension and studies have found that hypertension is often untreated in rural areas in Bangladesh [32], awareness building regarding consequences of water salinity and hypertension is necessary to prevent and control future health risks.

Our study also found increased hospital visit rates for diarrhea and abdominal pain due to drinking water salinity. Although there are very limited studies directly associating water salinity with diarrhea and abdominal pain, the local community in Bangladesh reported diarrhea, dysentery, and indigestion as some of the most common diseases they suffer from due to increased saline intake [26,33]. Diarrhea is particularly prevalent in children and infants who drink saline water and can be life threatening. 

Interestingly, our study revealed that people living in low salinity areas were more aware of health hazards of high drinking water salinity. A large number of people (76%) from high salinity areas did not change their water source and nearly 52% of them did not think drinking water salinity posed a significant health risk. This was contrary to previous community perception studies where locals believed that water salinity had impacted their daily lives and not only caused severe diarrhea, skin diseases, hypertension, and other health effects, but also additional hardships for women as they had to travel far to fetch clean water [26,33,34]. 

Despite threat of increasing water salinity in Bangladesh, the population remains largely unaware of the health hazards associated with the same. The risks involved have not been communicated to the people efficiently. Awareness is the essential first step in promoting risk-reducing behaviors. Future studies can focus on awareness building and educational interventions that may motivate the communities to switch to clean freshwater sources. In addition, there is vast potential for examining relationship between drinking water salinity and other health effects concerning CVD and beyond. 

Studies have found current strategies to cope with the increasing shortage of safe drinking water inadequate [35]. Different lifestyles, beliefs, and practices of people in Bangladesh demand that interventions be designed compatible with different communities [36]. Lack of capacity of the local government and weak government policies lead to failure of management of coastal polders, excessive shrimp farming, and/or over extraction of groundwater [6]. Existing government policies often do not involve the local governing bodies and non-governmental organisations (NGOs) that have a better understanding of and access to the local communities. Assistance provided by the NGOs is also reported as disproportionate with people in power receiving more aid than those who are not [35]. In order to design effective mitigation measures, government, and non-government organizations need to coordinate and work unbiased at the grassroots level to address the emerging water salinity issue with urgency [34].

### Potential Limitations 

There were several methodological limitations in our study. Our study used self-reported hospital visits, which might lead to misclassification of disease status. However, our interviewers asked additional questions including which hospital they were admitted to, symptoms of the disease, and/or prescriptions where available to confirm the health effect. So, the chances of misclassification are minimum and non-differential. 

We also lacked individual level information due to the pilot study design, which is another weakness in our study.

Dietary salt intake was not considered in our study, which might lead to an underestimation of daily salt intake. However, sodium consumption via drinking water in Bangladesh far exceeds that via daily food intake [37,38]. A study found that mean sodium levels in surface and ground water were nearly 516 mg/L [13]. This signifies a daily sodium intake of 1–1.5 g/day via drinking water alone (assuming a daily water intake of 2L). This is nearly 27 times the recommended daily sodium intake standard of 20 mg/L in drinking water set by US EPA. In another Bangladesh study, it was reported that nearly 50% of the young adults in coastal population were consuming sodium above the WHO recommended daily guideline value of 2 g/day, with tubewell water consumption alone contributing to nearly 1.7 g of daily sodium intake (assuming 2 L intake of drinking water) [39]. 

In our study, we reported hospital visits instead of health effects as a measure of health implications of drinking water salinity. Several previous studies have effectively used this proxy measure [11,40]. Focusing on hospital visits not only allows us to estimate the health effects that are caused due to drinking excessive sodium from water, but also gives an insight to the associated hospital and healthcare costs. 

Additionally, other confounding factors that might have caused the reported health effects were not considered. This might have led to an overestimation of the association between drinking water salinity and the health effects. However, our results are consistent with several previous studies that have reported relationships between increased intake of saline water and CVD, diarrhea, and abdominal pain.

Also, it has been seen that seasonality affects salinity levels, with the dry months leading to an increase in salinity. We did not consider the seasonal variation in water salinity levels, which is a limitation in our study. Larger studies can take this into account in the future. 

## 5. Conclusions

In our study, we have reported associations between elevated drinking water salinity and increased frequency of hospital visits for CVD, diarrhea, and abdominal pain, which increased with increasing quartiles of water salinity. Unexpectedly, we also found that the people exposed to high salinity did not perceive drinking water salinity to be a significant health risk. Since climate change is predicted to exacerbate water salinity levels, the benefits of alternative and low-cost adaptation strategies for providing freshwater such as rainwater harvesting, paying a nominal fee for safe water delivery, or technologically more advanced filtration systems for surface water use must be efficiently communicated to the people. Water salinity, if unchecked, will continue to rise and lead to a host of severe health effects and be a heavy burden on the people in the near future. Raising awareness regarding its impact on daily healthy lives and the possible coping mechanisms to obtain freshwater may motivate the communities to adapt to safe water consumption behaviors and also help the policymakers frame effective mitigation strategies to control drinking water salinity. 

## Figures and Tables

**Table 1:** Socio–demographic and exposure characteristics of participants.

Sociodemographic Characteristics	Low Salinity (*n* = 78) (Mean ± SD)	High Salinity (*n* = 79) (Mean ± SD)	*p*-Value
Age	42.0 ± 12.1	38.5 ± 9.9	0.050
Sex (male) (%, *n*)	98.7 (77)	97.5 (77)	0.239
Household size	5.5 ± 2.2	5.3 ± 1.5	0.38
BMI (Kg/m^2^)	25.8 ± 4.4	24.6 ± 4.3	0.075
Education (≥ elementary) (%, *n*)	57.9 (45)	43.0 (34)	0.066
Annual income (in 1000 Bangladesh Taka)	163.9 ± 127.3	145.5 ± 115.3	0.324
Smoking (%, *n*)	84.6 (66)	82.3 (65)	0.745
Water Salinity (mg/L)			
Drinking use(tubewell)	53.9 ± 49.2	1291.9 ± 1129.3	<0.0001 *
Both (Drinking and other use)	143.4 ± 25.8	856.8 ± 1082.5	0.136
Total Dissolved Solids (mg/L)			
Drinking use(tubewell)	73.6 ± 66.3	1610.0 ± 1355.4	<0.0001 *
Both (Drinking and other use)	193.1 ± 34.5	1073.4 ± 1313.2	0.130

**Table 2:** Frequency of three or more hospital visits in members of each household due to drinking water salinity in the past year.

Health Effects	Low Salinity and Total Dissolved Solids (TDS) (*n* = 78) (%, *n*)	High Salinity and TDS (*n* = 79) (%, *n*)	*p*-Value
Cardiovascular (CVD)	2.6 (2)	10.1 (8)	<0.05 *
Diarrhea	3.8 (3)	10.1 (8)	<0.05 *
Abdominal pain	3.8 (3)	13.9 (11)	<0.05 *
Gastric ulcer	20.5 (16)	21.5 (17)	0.989
Dysentery	7.7 (6)	11.4 (9)	0.256
Skin Diseases	9 (7)	8.9 (7)	1.000
Typhoid	5.1 (4)	10.1 (8)	0.452

Note: *p*-values are two sided and based on Chi-square test or Fisher’s exact test. The salinity category was determined based on the median drinking water salinity.

**Table 3:** Associations between categories of salinity exposure and hospital visits.

Reason for Visit	Salinity	OR (95% CI)
Cardiovascular (CVD)	Q1 (Reference)	1
	Q2	1.12 (0.21, 6.12)
	Q3	0.13 (0.01, 1.11)
	Q4	1.64 (0.25, 10.99)
Diarrhea	Q1 (Reference)	1
	Q2	0.43 (0.14, 1.38)
	Q3	0.34 (0.08, 1.49)
	Q4	1.75 (0.35, 8.75)
Abdominal pain	Q1 (Reference)	1
	Q2	0.35 (0.09, 1.48)
	Q3	2.30 (0.80, 6.72)
	Q4	3.64 (0.69, 19.24)

Note: Logistic models were adjusted for education and annual household income.
